# Hyperdense Aneurysm Visualized at the Occlusion Site During Thrombectomy: A Case Report

**DOI:** 10.7759/cureus.100302

**Published:** 2025-12-28

**Authors:** Ryosuke Kaneko, Hiroyuki Ikeda, Toshio Fujiwara, Shohei Yoshida, Minami Uezato, Masanori Kinosada, Yoshitaka Kurosaki, Masaki Chin

**Affiliations:** 1 Department of Neurosurgery, Kurashiki Central Hospital, Okayama, JPN

**Keywords:** aneurysm, case report, hidden aneurysm, hyperdense sign, mechanical thrombectomy

## Abstract

The occurrence of previously unrecognized aneurysms during mechanical thrombectomy for large-vessel occlusion poses a significant challenge. Aneurysms distal to the occluded vessel are often difficult to identify preoperatively despite the use of multiple advanced imaging modalities. However, important diagnostic clues may still be present even on plain CT scans. This case report describes a 79-year-old male who presented with acute left hemiparesis due to an occlusion of the right internal carotid artery. The aneurysm was identified intraoperatively but was not detected preoperatively. A retrospective review of the preoperative CT, however, demonstrated hyperdense findings that deviated from expected vascular anatomy. During thrombectomy, the aneurysm became apparent after deployment of the stent retriever, and the procedure was safely completed by withdrawing the stent retriever into the aspiration catheter while avoiding excessive mechanical stress on the aneurysm. This hyperdensity is believed to result from stagnation of blood flow within the aneurysm, caused by thrombus in the parent vessel. Successful recanalization was achieved without intracranial hemorrhage, and the patient demonstrated postoperative neurological improvement. Our findings suggest that careful review of preoperative imaging might have allowed earlier recognition. While hyperdense signs are generally considered indicative of thrombus, evaluating whether such findings conform to normal vascular anatomy may improve the preoperative detection of occult aneurysms.

## Introduction

Aneurysms have an estimated global prevalence of approximately 3% [1]. As their risk factors are similar to those of ischemic stroke [2], unrecognized aneurysms should be considered during thrombectomy. Aneurysm rupture during thrombectomy occurs in approximately 0.3 to 5.8% of cases [3,4]. However, aneurysms located distal to occluded vessels are difficult to identify preoperatively. To evaluate distal occlusion, contrast-enhanced flat-detector CT (FDCT) [5,6] and assessment of the capillary phase of contrast-enhanced CT [7] have been considered. However, additional clinically relevant information may be obtained from hyperdense signs on routinely acquired plain CT [8]. While differentiating aneurysm-related hyperdensity from typical thrombus-related hyperdensity can be challenging, we report a case in which a retrospective review enabled identification of an aneurysm concealed at the occlusion site, based on hyperdense signs recognized through detailed preoperative image interpretation.

This article was previously presented as a meeting abstract at the 16th AAFITN Congress in Bangkok, Thailand, on August 21-23, 2025. This case report details the clinical course of a single patient and is presented in accordance with the CARE guidelines. Ethical approval for this case report was not required as per institutional guidelines for retrospective case reports. Written informed consent was obtained from the patient for the publication of this case report and any accompanying images.

## Case presentation

History and examination

**Figure 1:**
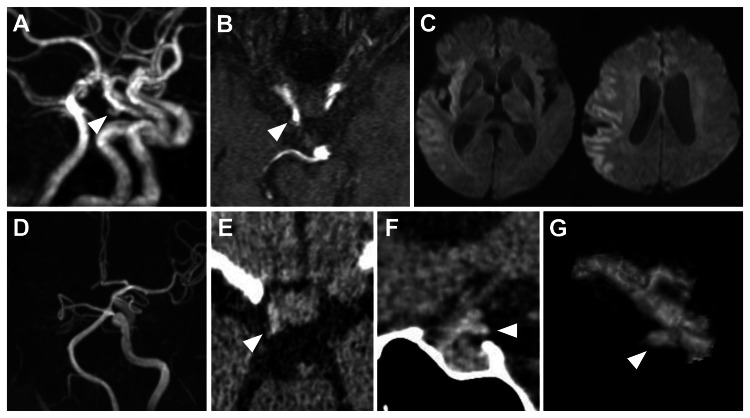
Preoperative images Three-dimensional (A) and axial (B) magnetic resonance angiography (MRA) performed 4 years ago showing a right internal carotid artery–posterior communicating artery (IC-PC) aneurysm (arrowhead). (C) Preoperative diffusion-weighted imaging (DWI) showing faint hyperintense areas in the right middle cerebral artery territory (DWI-Alberta Stroke Program Early Computed Tomography [CT] Score, 4 points). (D) Preoperative MRA showing right internal carotid artery occlusion. Preoperative plain axial (E) and sagittal (F) CT showing hyperdensity in the occluded vessel and the right IC-PC aneurysm area (arrowhead). (G) Reconstructed image showing hyperattenuated areas of hyperdensity on preoperative plain CT showing right IC-PC aneurysms (arrowhead) within the occluded internal carotid and middle cerebral arteries

A 79-year-old male patient was hospitalized at another institution for rehabilitation after an episode of pyelonephritis. In a retrospective review of the imaging, magnetic resonance angiography (MRA) performed four years earlier at our hospital had shown a 4-mm aneurysm in the right internal carotid artery (ICA) to the posterior communicating artery (IC-PC) junction (Figures 1A-1B). At the referring hospital, left hemiparesis was noted 90 minutes after the last known well time. Diffusion-weighted MRI (DW-MRI) performed 140 minutes after the last known well showed faint hyperintense areas in the right middle cerebral artery (MCA) territory, with a diffusion-weighted imaging Alberta Stroke Program Early CT Score (DWI-ASPECTS) of 4 points (Figure 1C). MRA showed occlusion of the right ICA (Figure 1D). The patient was transferred to our hospital 220 minutes after the last known well for endovascular thrombectomy. On arrival, the Glasgow Coma Scale (GCS) score was E3V3M6, with right conjugate gaze deviation, left hemiparesis, and dysarthria, and a National Institutes of Health Stroke Scale (NIHSS) score of 17 points. Head CT at our hospital showed hyperdense signs in the right ICA from the C2 segment to M1, as well as in the right IC-PC aneurysm area (Figures 1E-1G). Owing to the low ASPECTS score, tissue plasminogen activator was not administered, and thrombectomy was performed. At the start of the thrombectomy, the aneurysm at the right IC-PC was not recognized.

Endovascular surgery

**Figure 2:**
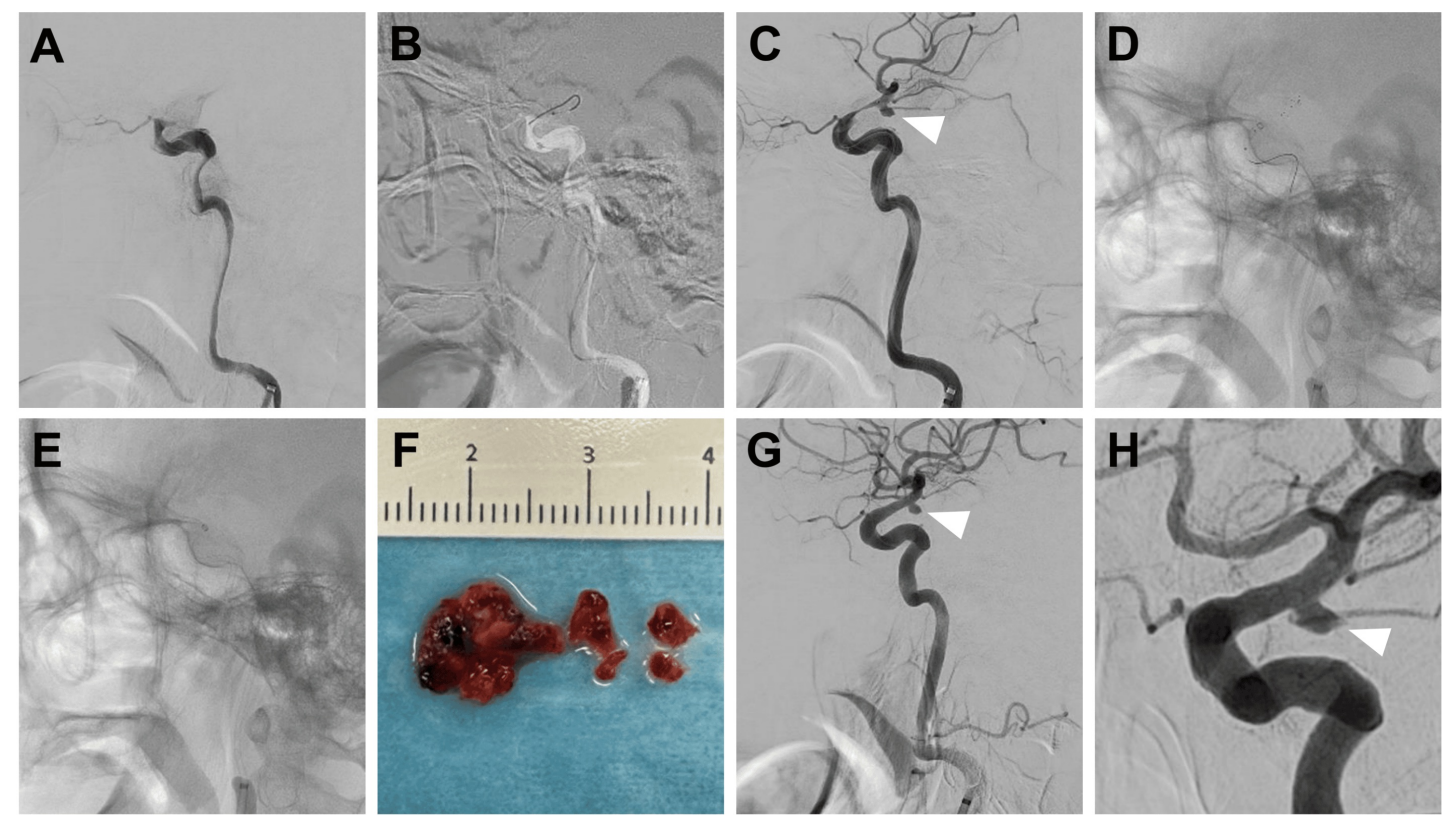
Intraoperative images (A) Preoperative right internal carotid arteriography showing the patency of the ophthalmic artery branch and occlusion of the internal carotid artery (ICA). (B) Lesion crossing of the occlusion site with a J-shaped microguidewire tip. (C) Right internal carotid arteriography after stent retriever deployment showing antegrade flow in the ICA and M1 segments and a right internal carotid artery–posterior communicating artery (IC-PC) aneurysm (arrowhead) within the occlusion site. (D) The aspiration catheter guided to the proximal end of the thrombus. (E) Ths stent retriever being retracted into the aspiration catheter. (F) Retrieved red thrombus. Post-thrombectomy right internal carotid arteriography lateral (G) and working angles (H) showing complete ICA recanalization and right IC-PC aneurysm (arrowhead)

Under local anesthesia, a 9-Fr Optimo (Tokai Medical, Kasugai, Japan) was placed in the cervical portion of the right ICA via the right femoral artery. Right internal carotid arteriography confirmed the patency of up to a branch of the ophthalmic artery (Figure 2A). The J-shaped Tracxcess (Terumo Neuro, Aliso Viejo, CA), Phenom 21 (Medtronic, Dublin, Ireland), and React 71 (Medtronic) were guided to perform lesion crossing at the occlusion site (Figure 2B). Angiography of the Phenom 21 guided to the proximal M2 superior trunk confirmed that the tip of the Phenom 21 was distal to the thrombus. A Solitaire X 4 mm × 40 mm (Medtronic) was deployed from the distal M1 to the ICA C2. Post-deployment right internal carotid arteriography from the React 71 showed antegrade flow in the thrombosed ICA and M1 segments and revealed a right IC-PC aneurysm within the occlusion site (Figure 2C). The React 71 was guided to the proximal end of the thrombus using the Solitaire X as the anchor (Figure 2D). To prevent the React 71 from advancing and causing mechanical stress on the aneurysm, the Solitaire X and Phenom 21 were pulled into the aspirating React 71 (Figure 2E). The aspirating React 71 was withdrawn, and a red thrombus was retrieved using both the Solitaire X and React 71 (Figure 2F). Post-thrombectomy right internal carotid arteriography showed complete recanalization of the ICA territory and the right IC-PC aneurysm (Figures 2G-2H).

Postoperative course

**Figure 3:**
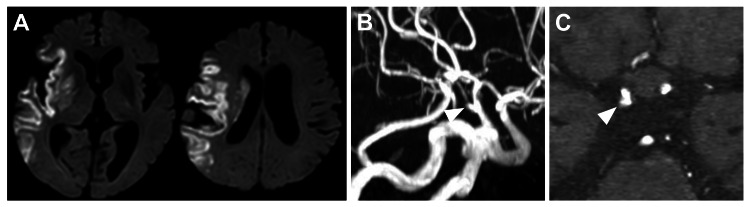
Postoperative images (A) Postoperative diffusion-weighted imaging showing cerebral infarction in the right middle cerebral artery territory and absence of intracranial hemorrhage. Postoperative three-dimensional (B) and axial (C) magnetic resonance angiography showing a right internal carotid artery–posterior communicating artery aneurysm (arrowhead)

MRI on postoperative day one showed cerebral infarction in the right MCA territory, but no intracranial hemorrhage (Figure 3A). MRA revealed a right IC-PC aneurysm (Figures 3B-3C). The consciousness level on postoperative day one was GCS E4V4M6, and the left upper and lower extremities improved to Manual Muscle Testing 3/5, with the National Institutes of Health Stroke Scale score improving to 9. Due to atrial fibrillation, direct oral anticoagulants were started on postoperative day four. The patient was transferred for rehabilitation on postoperative day 24, with a modified Rankin Scale (mRS) score of 4. The patient continued to have an mRS score of 4 at 90 days.

## Discussion

This patient had a right IC-PC aneurysm that had been identified on MRA four years before presentation. Furthermore, hyperdensity was present within the right IC-PC aneurysm on the head CT obtained immediately before surgery. However, the aneurysm was not recognized at the start of thrombectomy. Once the aneurysm was identified during thrombectomy, the procedure was safely completed by slowly retracting the stent retriever to prevent the aspiration catheter from advancing into the aneurysm and causing excessive mechanical stimulation. Although prior case reports have described occult aneurysms discovered during endovascular treatment, we believe that emphasis on preoperative hyperdense imaging findings represents a novel aspect of the present report.

The prevalence of aneurysms is estimated to be approximately 3% [1]. However, because their risk factors overlap with those of ischemic stroke [2], the possibility of encountering unrecognized aneurysms during thrombectomy should be routinely considered. Aneurysms have been reported to coexist in approximately 5% [4] and 3.7 to 6.6% [9] of acute ischemic stroke cases, with intraoperative ruptures occurring in 2.8% [4] and 0.3 to 5.8% [3] of cases. The MCA is reported as the most common location [10]. Although some reports indicate that ICA aneurysms are relatively rare, others have found that up to 47.6% occur in the ICA [4].

For preoperative identification, contrast-enhanced FDCT [5,6] and assessment of the capillary phase of contrast-enhanced CT [7] have been proposed to evaluate distal occlusions. However, even without additional imaging, hyperdensity signs on CT and T2* sequences on MRI remain useful [10]. In particular, findings that exceed the expected diameter of the parent artery warrant careful attention [8]. In a retrospective review, the most important finding in this case was the preoperative hyperdensity sign, which may have been more easily recognized had sagittal views been assessed. In clinical practice, imaging interpretation is often limited to axial views only. There are inherent limitations in drawing general conclusions from a single case. However, adopting a three-dimensional approach and focusing on findings that deviate from the expected course and diameter of the parent vessel may significantly improve the detection of hidden aneurysms.

In this case, because the entire aneurysm was clearly visualized on angiography after stent retriever deployment, it was unlikely that the preoperative hyperdensity sign indicated thrombus formation within the aneurysm. Originally, hyperdensity signs were reported as markers of thrombus at the occlusion site [11]. Although the Hounsfield unit (HU) scale is a relative indicator, the CT absorption value of blood in normal circulation ranges from 40 to 43 HU (average 41.3), whereas occlusive thrombi demonstrate values of 47 to 61 HU (average 54.0). In our case, the HU value was 61. The thrombus becomes hyperattenuated because plasma is expelled from the thrombus, increasing the local hematocrit [12,13]. Consequently, blood under conditions of markedly slow or stagnant flow may also appear hyperattenuated on CT [14]. Previous reports have suggested that changes in blood flow due to occlusion or recanalization of the parent or proximal vessels of aneurysms can cause sudden thrombosis within aneurysms [15,16]. In this case, hyperdensity was attributed to blood flow stagnation within the aneurysm caused by thrombus formation in the parent vessel at the aneurysm site. Importantly, the presence of aneurysm-like hyperdensity does not necessarily indicate the presence of a thrombus extending into the aneurysm.

Regarding the technique, previous reports have described cases in which aneurysms were not recognized preoperatively and subsequently ruptured [5,6], with stent retriever retraction or microwire perforation implicated as possible causes. The J-shaped microguidewire (MG) tip and ADAPT (A Direct Aspiration first Pass Technique) are considered safer alternatives [17]. However, even with a J-shaped MG tip, perforation can still occur in terminal-type aneurysms. In this case, lesion crossing was performed with a J-shaped MG tip at the occlusion site, where a side-wall type aneurysm was present. During stent retriever retraction, the stent retriever was drawn back into the aspiration catheter and positioned at the proximal end of the thrombus. This approach limited vascular deviation and minimized mechanical stress on the aneurysm.

Care was taken to prevent the aspiration catheter from advancing beyond this point and causing excessive mechanical stress on the aneurysm. Nevertheless, if an aneurysm at the occlusion site is recognized preoperatively, ADAPT with an aspiration catheter guided to the proximal end of the thrombus, without advancing the microcatheter or MG through the occlusion site, may represent the safest option. For side-wall-type aneurysms, lesion crossing without exerting stress on the aneurysm may be achieved with a J-shaped MG tip. If an aneurysm is detected during angiography after stent retriever deployment, a combined technique in which an aspiration catheter is guided to the proximal thrombus margin may further enhance procedural safety by reducing mechanical stress on the aneurysm during stent retriever retraction.

## Conclusions

This case was unusual in that an aneurysm within the occluded vessel presented as a hyperdense sign on preoperative CT but was only recognized during thrombectomy. While hyperdense signs are generally interpreted as markers of thrombus, they may also provide valuable information for the preoperative identification of aneurysms hidden distal to the occlusion site. A more comprehensive interpretation, particularly one that evaluates whether hyperdense findings deviate from the expected morphology, trajectory, or luminal dimensions of normal vascular structures, may substantially improve the preoperative detection of hidden aneurysms.
